# Effect of rTMS intervention on upper limb motor function after stroke: A study based on fNIRS

**DOI:** 10.3389/fnagi.2022.1077218

**Published:** 2023-01-13

**Authors:** Jing Ni, Wei Jiang, Xueyang Gong, Yingjie Fan, Hao Qiu, Jiaming Dou, Juan Zhang, Hongxing Wang, Chunguang Li, Min Su

**Affiliations:** ^1^Department of Physical Medicine and Rehabilitation, Dushu Lake Hospital Affiliated of Soochow University, Suzhou, Jiangsu, China; ^2^Department of Physical Medicine and Rehabilitation, Jiangsu Rongjun Hospital, Wuxi, Jiangsu, China; ^3^Department of Physical Medicine and Rehabilitation, Wuxi International Tongren Rehabilitation Hospital, Wuxi, Jiangsu, China; ^4^Institute of Rehabilitation Soochow University, Suzhou, Jiangsu, China; ^5^First Affiliated Hospital of Soochow University, Suzhou, Jiangsu, China; ^6^Department of Rehabilitation Medicine, Zhongda Hospital Southeast University, Nanjing, Jiangsu, China; ^7^The Key Laboratory of Robotics and System of Jiangsu Province, School of Mechanical and Electric Engineering, Soochow University, Suzhou, Jiangsu, China

**Keywords:** stroke rehabilitation, upper limb motor function, rTMS, fNIRS, brain functional network

## Abstract

**Background:**

Stroke is a disease with a high fatality rate worldwide and a major cause of long-term disability. In the rehabilitation of limb motor function after stroke, the rehabilitation of upper limb function takes a long time and the recovery progress is slow, which seriously affects the patients’ self-care ability in daily life. Repeated transcranial magnetic stimulation (rTMS) has been increasingly used to improve limb dysfunction in patients with stroke. However, a standardized reference for selecting a magnetic stimulation regimen is not available. Whether to increase the inhibition of the contralateral hemispheric motor cortex remains controversial. This study has evaluated the effects of different rTMS stimulation programs on upper limb function and corresponding brain functional network characteristics of patients with stroke and sought a new objective standard based on changes in brain network parameters to guide accurate rTMS stimulation programs.

**Method:**

Thirty-six patients with stroke were selected and divided into control group and treatment group by number table method, with 18 patients in each group, and 3 patients in the control group were turned out and lost due to changes in disease condition. The treatment group was divided into two groups. TMS1 group was given 1 Hz magnetic stimulation in the M1 region of the contralesional hemisphere +10 Hz magnetic stimulation in the M1 region of the affected hemisphere, and the TMS2 group was given 10 Hz magnetic stimulation in the M1 region of the affected hemisphere. The control group was given false stimulation. The treatment course was once a day for 5 days a week for 4 weeks. The Fugl-Meyer Assessment for upper extremity (FMA-UE) sand near-infrared brain function were collected before treatment, 2 weeks after treatment, and 4 weeks after treatment, and the brain function network was constructed. Changes in brain oxygenated hemoglobin concentration and brain network parameters were analyzed with the recovery of motor function (i.e., increased FMA score). Meanwhile, according to the average increment of brain network parameters, the rTMS stimulation group was divided into two groups with good efficacy and poor efficacy. Network parameters of the two groups before and after rTMS treatment were analyzed statistically.

**Results:**

(1) Before treatment, there was no statistical difference in Fugl-Meyer score between the control group and the magnetic stimulation group (*p* = 0.178).Compared with before treatment, Fugl-Meyer scores of 2 and 4 weeks after treatment were significantly increased in both groups (*p* <0.001), and FMA scores of 4 weeks after treatment were significantly improved compared with 2 weeks after treatment (*p* < 0.001). FMA scores increased faster in the magnetic stimulation group at 2 and 4 weeks compared with the control group at the same time point (*p* <0.001).TMS1 and TMS2 were compared at the same time point, FMA score in TMS2 group increased more significantly after 4 weeks of treatment (*p* = 0.010). (2) Before treatment, HbO2 content in healthy sensory motor cortex (SMC) area of magnetic stimulation group and control group was higher than that in other region of interest (ROI) area, but there was no significant difference in ROI between the two groups. After 4 weeks of treatment, the HbO2 content in the healthy SMC area was significantly decreased (*p* < 0.001), while the HbO2 content in the affected SMC area was significantly increased, and the change was more significant in the magnetic stimulation group (*p* < 0.001). (3) In-depth study found that with the recovery of motor function (FMA upper limb score increase ≥4 points) after magnetic stimulation intervention, brain network parameters were significantly improved. The mean increment of network parameters in TMS1 group and TMS2 group was significantly different (*χ*^2^ = 5.844, *p* = 0.016). TMS2 group was more advantageous than TMS1 group in improving the mean increment of brain network parameters.

**Conclusion:**

(1) The rTMS treatment is beneficial to the recovery of upper limb motor function in stroke patients, and can significantly improve the intensity of brain network connection and reduce the island area. The island area refers to an isolated activated brain area that cannot transmit excitation to other related brain areas. (2) When the node degree of M1_Healthy region less than 0.52, it is suggested to perform promotion therapy only in the affected hemisphere. While the node degree greater than 0.52, and much larger than that in the M1_affected region. it is suggested that both inhibition in the contralesional hemisphere and high-frequency excitatory magnetic stimulation in the affected hemisphere can be performed. (3) In different brain functional network connection states, corresponding adjustment should be made to the treatment plan of rTMS to achieve optimal therapeutic effect and precise rehabilitation treatment.

## 1. Introduction

Stroke has high morbidity and mortality worldwide and is also one of the most common causes of neurological disability (Roger et al., 2012; Delorme et al., 2019). More than two-thirds of patients with stroke have impaired upper limb motor function (Nakayama et al., 1994). Their independent daily living activities are affected, limiting patients’ return to family and society, bringing psychological and economic burdens to patients and their families, and causing a certain degree of social burden. At present, the rehabilitation of upper limb function is one of the difficulties in rehabilitation patients with stroke (Bertani et al., 2017). Rehabilitation methods such as exercise therapy and occupational therapy can restore the motor function of the upper limbs. In addition to traditional rehabilitation methods, in recent years, noninvasive brain stimulation, including transcranial magnetic stimulation (TMS), transcranial direct current stimulation (tDCS), and other new methods, have been considered influential in restoring upper limb motor function (Van Lieshout et al., 2019).

Repetitive TMS (rTMS) is a noninvasive and painless method of regulating cortical excitability. Cortical excitability is increased by high-frequency rTMS and inhibited by low-frequency rTMS (Pascual-Leone et al., 1998). At present, two theoretical models for the clinical application of rTMS in stroke exercise rehabilitation are the bilateral hemispheric competition model of “inhibition of affected hemisphere and excitation of contralesional hemisphere” (Wassermann et al., 1991; Ferbert et al., 1992; Corti et al., 2012; Grefkes and Fink, 2014) and the compensation model of the residual brain area and the contralesional hemisphere (Ward, 2011). Given that these two theoretical models are inconsistent in guiding TMS therapy, consensus has not been reached whether to use inhibitory magnetic stimulation in the contralesional hemisphere (Long et al., 2018). Some studies showed that rTMS can inhibit the activation of the contralateral cortex of the lesion, thus improving the motor function of the affected limb (Shimizu et al., 2002; Khedr et al., 2009; Du et al., 2019), but not all patients show good recovery effects. Studies showed that high-frequency excitatory magnetic stimulation alone on the affected side can also benefit (Khedr et al., 2010; Sasaki et al., 2017). Other studies found that the good effect after inhibition in the contralesional hemisphere depends on the degree of corticospinal tract injury in the affected hemisphere (Stinear et al., 2007; Lotze et al., 2012). Inhibition of magnetic stimulation of the contralesional hemisphere in patients with severe corticospinal tract injury fails to achieve the expected efficacy，while inhibitory magnetic stimulation of the contralesional hemisphere in patients with mild corticospinal tract injury has improved effect (Málly and Dinya, 2008; Frost et al., 2017; Lim et al., 2020; Liu et al., 2021; Zolkefley et al., 2021). However, even with diffusion tensor magnetic resonance imaging techniques, the extent of corticospinal tract injury in each patient is difficult to detect. Therefore, a simple method is needed to characterize the level of hemispheric connectivity and develop an accurate brain function treatment plan.

In recent years, with the continuous progress of brain function research, brain functional network connection is gradually known and has gradually become a research hotspot of brain-related diseases (Lubrini et al., 2018; Guggisberg et al., 2019; Jalilianhasanpour et al., 2019; Klaassens et al., 2019; Salvalaggio et al., 2020). By analyzing the characteristics of brain functional networks, some progress has been made in the research on brain dysfunction and its rehabilitation mechanism (Chen and Schlaug, 2013; Lefebvre et al., 2017; Hordacre et al., 2018; McCambridge et al., 2018). Currently, common brain imaging techniques include positron emission tomography (PET), magnetic resonance imaging (MRI), functional MRI (fMRI), electroencephalography (EEG), and functional near-infrared spectroscopy (fNIRS). In PET, MRI, and fMRI, subjects are required to remain still during the test (Sakudo, 2016). This situation is not good for detecting brain activity especially locomotion-related brain activity. Also, the imaging equipment is very expensive. Therefore, these techniques have certain limitations in clinical application. EEG has high time resolution and low cost. However, due to its low spatial resolution, EEG cannot accurately locate active brain regions, so its clinical application is limited.fNIRS has relatively little restriction on subjects’ body movements, supports continuous testing over long periods of time, and is inexpensive. The blood oxygen information detected by fNIRS can reflect the neural activity state of the corresponding brain region，and can construct the brain network through the blood oxygen information, which can guide the brain functional stimulation therapy. fNIRS also has certain limitations. Given its technical characteristics, deep brain regions are difficult to detect through fNIRS, and the structural information of the brain is impossible to obtain due to limited spatial resolution (Tamashiro et al., 2019).

The purpose of this study was using fNIRS technology to monitor cerebral oxygenated hemoglobin content, construct brain functional network characteristics in patients with stroke, and provide an appropriate treatment plan for rTMS. The rTMS technique is often applied for the treatment of upper limb dysfunction after stroke and is used before routine rehabilitation. fNIRS is used to monitor the brain activity during upper limb movement before and after rTMS, and the functional brain network is established on the basis of the information of cerebral oxygenated hemoglobin. The Fugl-Meyer Assessment for upper extremity (FMA-UE) scale is used to evaluate the corresponding upper limb motor function recovery progress. The effect of rTMS treatment is evaluated by changes in functional brain network and FMA-UE score before and after rTMS treatment, respectively. This study was aimed to seek out a novel method to obtain an appropriate therapeutic schedule as rTMS on account of brain network characteristics. Therefore, the brain functional networks before and after rTMS treatment are compared with each other, which was divided into two groups with good and poor treatment effects. According to the results of fNRIS, the stimulation site, frequency and intensity of rTMS should be determined to establish the best treatment plan to improve the rehabilitation efficiency and speed up the process of functional recovery of patients.

## 2. Materials and methods

### 2.1. Participants

A total of 80 patients with stroke admitted to the Department of Rehabilitation Medicine of the First Affiliated Hospital of Soochow University from January 2019 to January 2020 were screened for this study. Patients were screened in accordance with the inclusion and exclusion criteria, and 36 patients were included in this study. Patients with (1) unilateral hemiplegia secondary to a stroke confirmed by MRI or computer tomography; (2) stable vital signs, in conscious state, and examination can be conducted; (3) onset to the course of 1 month; (4) and Brunnstrom stage II-IV of the upper extremity were included. Patients (1) with diseases with severe or unstable clinical conditions; (2) the patients had other neurological illness in addition to stroke; (3) who were unable to understand test instructions and complete test tasks due to severe cognitive and communication disorders; (4) with pacemaker or metal implant or have a skull defect; and (5) additional contraindications for rTMS mentioned in the guidelines for use issued in 2020 were excluded (Rossi et al., 2021).

**Figure 1:**
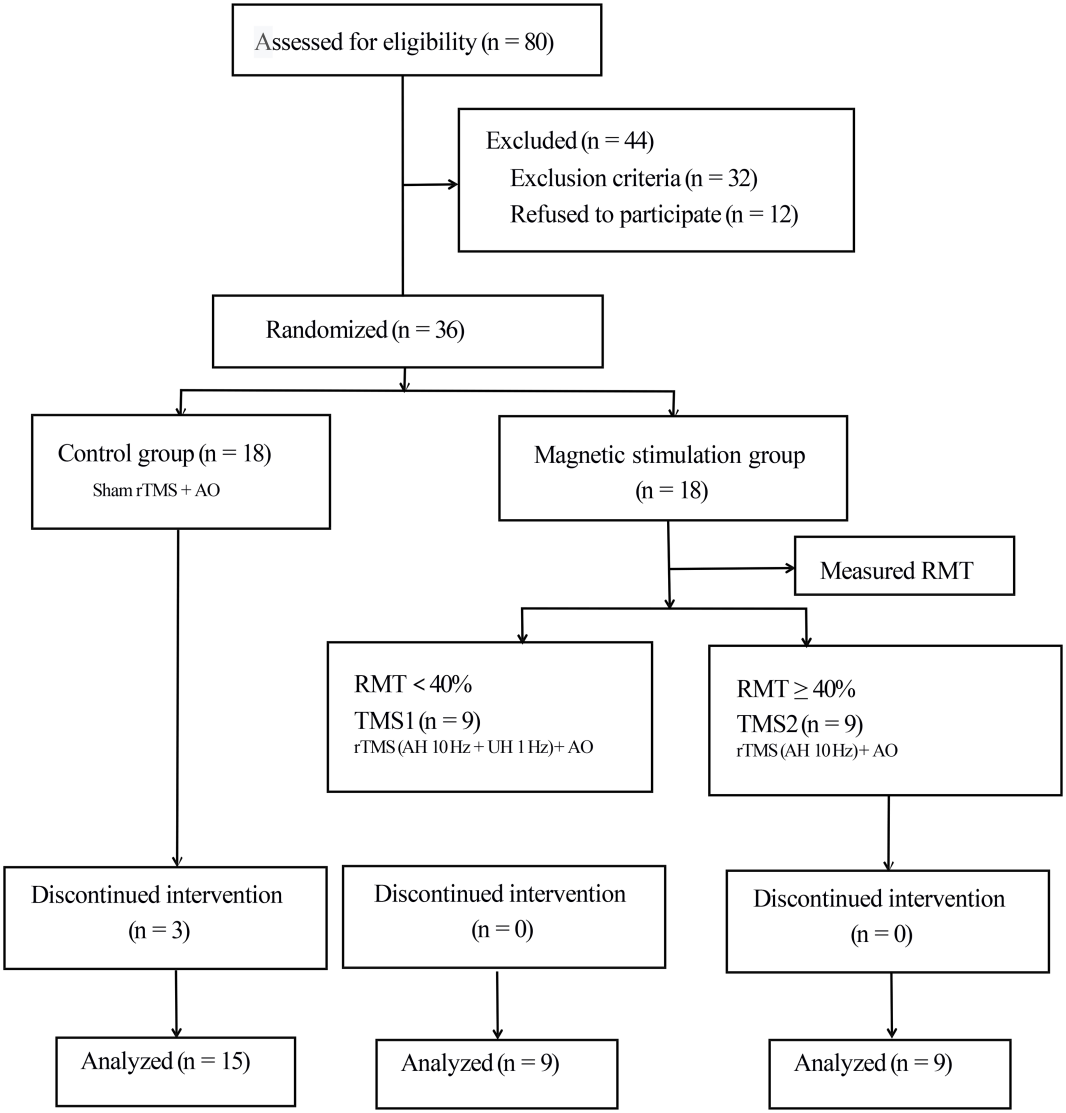
Consolidated Standards of Reporting Trials flow diagram. rTMS, repetitive transcranial magnetic stimulation; AO, action observation; AH, affected hemisphere; UH, unaffected hemisphere; RMT, resting motor threshold.

This study was conducted following the ethical standards in the 1975 Declaration of Helsinki and approved by the Internal Review Committee of Dushu Lake Hospital affiliated with Soochow University. Each participant signed an informed consent form. The trial was registered at the Chinese Clinical Trial Registry (CHICtr-IOR-14005394) and reported in accordance with the Integrated Standard for Trial Reporting Group guidelines. The participant flow chart is shown in Figure 1.

We divided 36 participants into the control and magnetic stimulation groups. The treatment group was further divided into two groups in accordance with the measured resting motor threshold (RMT; Rossini et al., 2015). In the control group, three patients were discharged and lost due to the request of their family members to be transferred to other hospitals for treatment, but the data lost did not influence the subsequent statistical analysis. A total of 33 subjects (24 males and 9 females) completed the experiment. The demographic data and clinical history of patients are summarized in Table 1.

**Table 1:** Demographic data and clinical history of the patients.

Patient	Group^a^	Sex^b^	Age (years)	Type of stroke	Brain lesion side^c^	FMA0^d^	FMA1^e^	FMA2^f^
1	1	M	53	infarction	**R**	16	21	26
2	1	M	51	infarction	**R**	23	29	34
3	1	M	53	infarction	**R**	22	30	35
4	1	F	54	infarction	**R**	21	24	38
5	1	M	58	hemorrhage	**L**	20	30	37
6	1	M	51	infarction	**R**	22	29	33
7	1	M	65	infarction	**L**	25	26	38
8	1	M	51	infarction	**L**	23	37	40
9	1	M	60	hemorrhage	**L**	18	22	38
10	1	F	64	hemorrhage	**R**	23	28	36
11	1	M	53	hemorrhage	**R**	20	22	38
12	1	M	54	Infarction+	**L**	24	32	37
13	1	M	68	hemorrhage	**R**	20	22	39
14	1	M	56	Infarction	**L**	20	29	34
15	1	F	67	hemorrhage	**R**	24	26	31
16	3	M	51	Infarction+	**Brainstem**	18	36	43
17	3	M	69	hemorrhage	**R**	24	32	44
18	2	M	57	Infarction	**R**	26	31	31
19	3	M	69	hemorrhage	**L**	15	36	40
20	2	M	67	hemorrhage	**L**	21	38	39
21	2	M	71	hemorrhage	**L**	19	35	36
22	2	M	50	Infarction	**R**	26	34	43
23	2	M	64	Infarction	**R**	24	31	35
24	2	F	61	Infarction	**L**	22	32	44
25	3	M	66	hemorrhage	**R**	27	34	44
26	3	M	67	Infarction	**L**	26	30	49
27	3	M	55	Infarction	**L**	22	35	40
28	2	M	54	Infarction	**R**	25	34	34
29	3	M	56	hemorrhage	**L**	19	37	44
30	3	F	52	Infarction	**R**	22	33	44
31	2	M	63	hemorrhage	**L**	28	37	39
32	3	M	51	Infarction	**R**	26	39	42
33	2	M	53	hemorrhage	**L**	22	34	43

a Group: 1 = control group, 2 = TMS1, 3 = TMS2.

b M, male; F, female.

c L, left; R, right.

d FMA-UE (score/66). FMA0 = before treatment.

e FMA1 = after treatment for 2 weeks.

f FMA2 = after treatment for 4 weeks.

### 2.2. Procedure and follow-up

Patients in both groups received routine rehabilitation training, including the Bobath technique, occupational therapy and AO therapy, and completed by licensed rehabilitation therapists for 40–60 min daily for 5 days a week for four weeks (Shah et al., 1986; Lennon and Ashburn, 2000; Fu et al., 2017; Guiu-Tula et al., 2017). The FMA-UE scale was determined by medical practitioners before treatment, 2 weeks after treatment, and 4 weeks after treatment (Gladstone et al., 2002). The fNIRS test was performed before treatment and 4 weeks after treatment.

#### 2.2.1. rTMS stimulation intervention

Each patient was treated with rTMS by using the MagPro R30 magnetic stimulator (McF-b65, water-cooled 8 type coil, Medtronic, Dublin, Ireland). The inner diameter of the single coil was 7.5 cm, and the peak stimulation intensity was 3 Tesla. On account of rTMS technique is mainly used to directly stimulate the cortex. This technique can improve the efficiency and effect of rehabilitation therapy if done before routine rehabilitation therapy, and can further consolidate and strengthen the excitability of cortex through conventional rehabilitation therapy. To sum up, the rTMS technique was used before routine rehabilitation in this study. For the abductor pollicis brevis muscles of healthy limbs, the minimum magnetic stimulus intensity that triggered a motor-evoked potential higher than 50 μV was the resting motion threshold (RMT) in 5 out of 10 stimuli (Ozdemir et al., 2021). RMT was measured on the same day after the first fNIRS test. Given that individuals had different RMTs, the energy level of treatment was determined on the basis of each patient’s RMT (Di Pino et al., 2014). The intensity of magnetic stimulation was set to 100% of RMT (Rossi et al., 2021).

Selection of stimulation site: fiber cap positioning in the EEG 10/20 system was adopted. The positioning cap was used to select the dominant area of hand function in the primary motor area (M1 area) of the healthy/affected side (generally located around the C3, C4 sites of the fiber cap). The best stimulation point was detected by slightly moving the stimulation coil during TMS assessment. In this study, high-frequency 10 Hz rTMS stimulation was performed on the M1 region of each patient’s affected hemisphere. The excitatory sequence consisted of 1,200 pulses. If RMT was less than 40% (Rossini et al., 2015), inhibitory 1 Hz magnetic stimulation was added to the M1 region of the contralesional hemisphere (Mineo et al., 2017; Du et al., 2018). The suppression sequence consisted of 120 pulses, which was repeated 10 times at 20 s intervals. Treatments were administered once a day, 5 days a week, for 4 weeks.

The treatment group was divided into two groups. In the TMS1 group, the M1 region of the contralesional hemisphere was subjected to a frequency of 1 Hz to suppress magnetic stimulation, and the M1 region of the affected hemisphere was treated at a frequency of 10 Hz to promote magnetic stimulation. In the TMS2 group, the M1 region of the affected hemisphere was subjected to a frequency of 10 Hz to promote magnetic stimulation. Sham stimulation was given to the control group, and the stimulation sites and parameters were the same as those of the TMS2 group, but the coil was perpendicular to the skull.

#### 2.2.2. fNIRS acquisition

**Figure 2:**
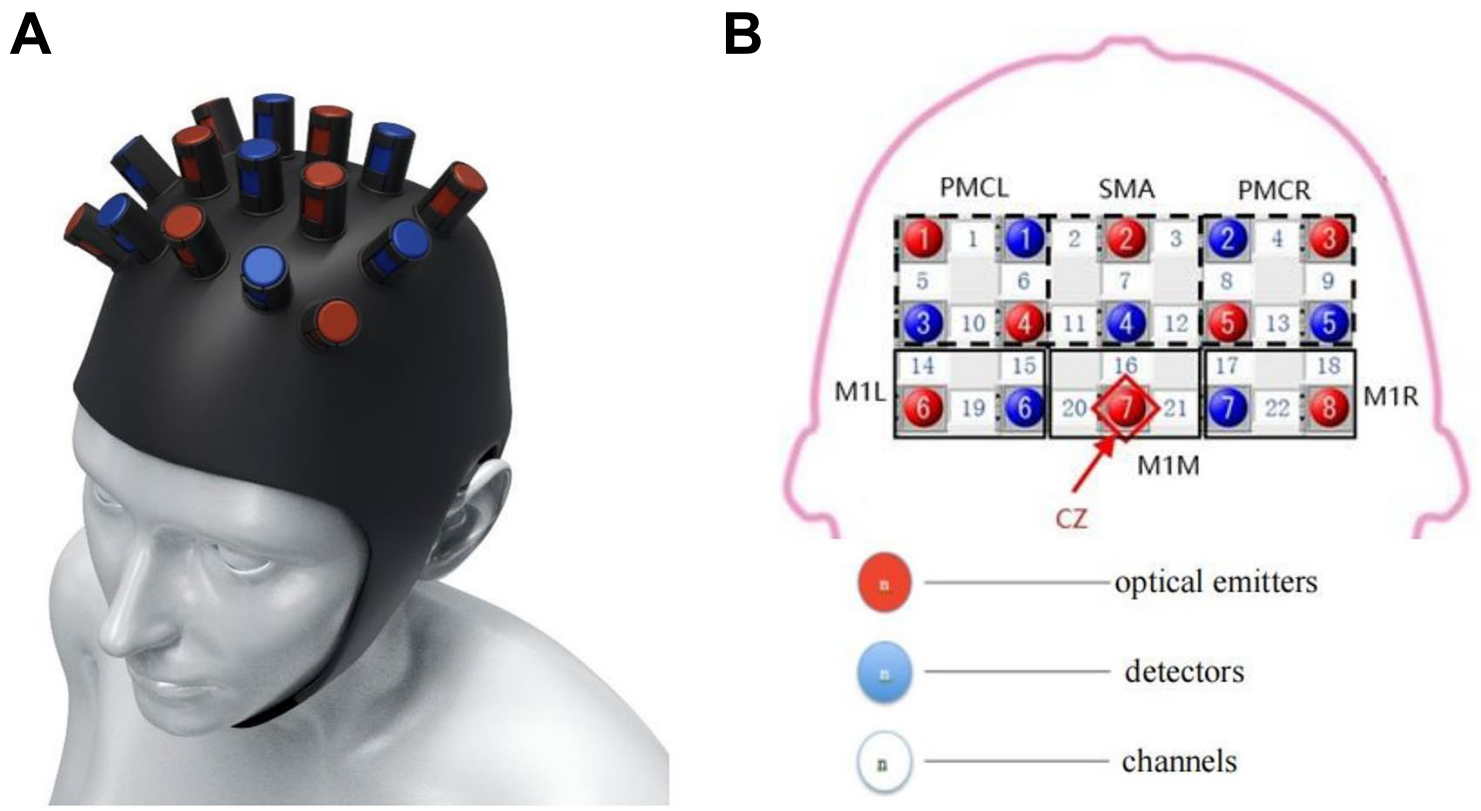
**(A)** Fiber cap positioning in the EEG 10/20 system. **(B)** Channel coverage in each ROI area.

Changes in oxyhemoglobin (oxyHb), deoxy-Hb, and total Hb concentrations were measured using the Force-3,000 near-infrared functional brain imager (Shimadzu Corporation, Kyoto, Japan). The sampling period of the hemoglobin signal was 0.13 s. A 3 × 5 top cover flash holder was used (Figure 2). In accordance with the 10–20-electrode system (Homan et al., 1987), emitter 7 was placed at the Cz vertex. Channels 19 and 22 were located at C3 and C4, respectively. Test channels were represented by numbers 1–22. Brain functional areas included bilateral premotor cortex (PMC; channels 1, 5, 6, and 10 on the left and channels 4, 8, 9, and 13 on the right), bilateral supplementary motor area (SMA; channels 2 and 11 on the left and channels 3 and 12 on the right), bilateral sensorimotor area (channels 14, 15, 19, and 20 on the left and channels 17, 18, 21, and 22 on the right). All regions are related to motor function and play an essential role in cognitive processing and motor control (Rehme and Grefkes, 2013; Wei et al., 2017; Cristofori et al., 2019).

#### 2.2.3. Motor tasks

**Figure 3:**
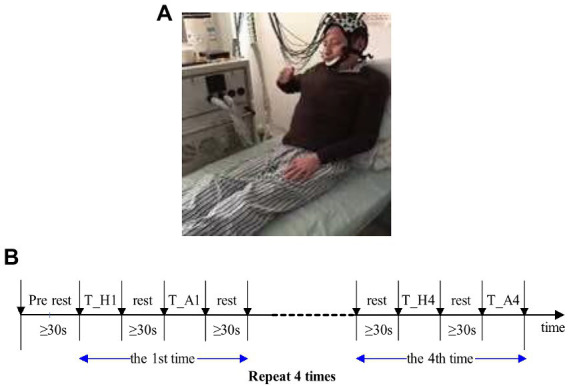
**(A)** Experimental diagram. **(B)** Sequence diagram of finger-to-nose test. T_H, motor task of the healthy side of the upper limb; T_A, motor task of the upper limb. Patients with muscle strength level below 3 were unable to complete the tasks so they should focus on completing the finger and nose test in motor imagination.

During the fNIRS test, patients were required to perform the finger-to-nose test (Figure 3A). The patient’s arms were placed on either side of the body or remained in a horizontal position in front of the body. Which happened naturally at rest. In the exercise task, the patient was first instructed to extend and straighten one arm, then bended the arm with the index finger pointing to the nose, and finally slowly returned the arm to its original position. After confirming that the patient understood the task activity, the patient with limited mobility was instructed to complete the finger-to-nose task as far as possible to reach the maximum restricted position and then slowly move the limb back to the original position. The beginning and end of each motor task were controlled by the patient. This phenomenon reduced changes in brain information caused by external stimuli (visual or auditory) and increased the reliability of brain hemoglobin information, which represented the motor function.

The sequence diagram of the finger and nose test is shown in Figure 3B. Each task period was alternated with rest periods. Patients were not allowed to count although sufficient rest time was guaranteed, i.e., not less than 30 s. The finger and nose test was completed continuously with the upper limb, and the first task began with the healthy limb. Patients tried to slow down the process of the healthy limb to motivate the affected limb, and task completion took about 8–9 s for each limb. The healthy and affected limbs were performed once separately as a set of tasks, and each set of tasks was repeated four times, that is, eight finger and nose trials. Before the experiment began, patients practiced 2 to 3 times to familiarize themselves with the movements and rest periods of the task. At the beginning and end of each exercise task, a marker was labeled with the FORIE-3000 by the experimental operator, and the marker was recorded simultaneously with the hemoglobin information. Alternate tasks between the affected and healthy limbs were performed to reduce the affected limb fatigue caused by repeated movement and the effect of continuous mechanical repetition on brain information.

### 2.3. Data processing

#### 2.3.1. Motor function assessment methods

The Fugl-Meyer Assessment for upper extremity (FMA-UE) scale was used to assess the upper limb motor function of the affected side of the two groups before and after treatment. As mentioned previously, medical practitioners performed the score of the FMA-UE before treatment, 2 weeks after treatment, and 4 weeks after treatment. The FMA-UE includes includs 9 items and 33 items of reflex, shoulder, elbow, wrist and hand, with a grade of 3 (0–2 points) and a total score of 66 points (Hijikata et al., 2020). Patients with higher scores indicate better function.

#### 2.3.2. fNIRS data processing

First, the collected signals by the force-3,000 near-infrared functional brain imager were filtered at 0.01–0.50 Hz to remove the interference of high-frequency noise and slow drift. Second, the optical signal was solved into the variation range of HbO2 and Hb concentration. Changes in HbO2 concentration between active and rest phases were recorded during the four tasks. The mean value of HbO2 concentration changes was calculated to avoid the randomness of the measurement signal and obtain accurate and stable experimental data. Then, changes in oxygenated hemoglobin concentration were analyzed statistically. All analyses were performed using data corresponding to the motor task of the affected limb. All the 22 channels were divided into nonlesional and lesional hemisphere. Then the hemoglobin signal of nonlesional and lesional hemisphere were used to calculate Laterality Index. Channel information from the left and right hemispheres was averaged and the corresponding average was used to calculate laterality. The left hemisphere includes channels 1, 2, 5, 6, 10, 11, 14, 15, 19, 20 and the right hemisphere includes channels 3, 4, 7, 8, 9, 12, 13, 16, 17, 18, 21, 22. We calculated the laterality index (LI; Balconi et al., 2015) for each trial to represent whether the cortical regions activated during each exercise were in the lesional or nonlesional hemispheres. LI was defined as: (∆Oxy-Hb in Affected Hemisphere − ∆Oxy-Hb in Unaffected Hemisphere)/(∆Oxy-Hb in Affected Hemisphere + ∆Oxy-Hb in Unaffected Hemisphere). The measured HbO concentration (∆OXY-Hb) refers to the change value of HbO during each exercise relative to the value measured in the resting state. The range of LI values is −1 to 1. −1 represents the activation of the nonlesional hemisphere, and 1 represents the activation of the lesional hemisphere (Delorme et al., 2019).

The wavelet transform coherence algorithm was used to calculate the coherence (Holper et al., 2012) of 22 channels in the center frequency of 0.04 Hz. The Morlet wavelet was selected as the mother wavelet. A channel was defined as a node. An adjacency matrix with 22 × 22 coherence coefficients (Coeff) was obtained for each motion task. A confidence level test was performed on the adjacency matrix to set the threshold. The consistency coefficient was quantified as “1” when confidence was 0.9. Otherwise, the consistency coefficient was “0.” The threshold value of 0.9 was selected on the basis of the actual results of the adjacency matrix, in which the links of some patients were reserved.

The Coeff represents the degree to which brain channels are connected relative to limb movements. The network parameters “node degree” (ND) and “clustering coefficient” (CC Sun et al., 2021) were calculated for the six regions and the whole test range (six regions together). ND and CC were selected to reflect the data communication capability of each functional area. The formula of ND is shown in Eqs. (1) and (2):


(1) $$K=\Phi_{degree}\;\left(M_{adj}\right)=\frac{1}{N}\overset{N}{\underset{i=1}{\sum }}K_{i}$$

(2) $$K_{i}=\overset{N}{\underset{j=1,j\neq i}{\sum }}M_{adj}\left(i,j\right)\;$$

N is the number of nodes in a region, K is the number of edges of node I, and Ki is the ND of the corresponding region. Compared with CC, ND has a normalized range of 0–1. The calculation formula of CC is shown in Eqs. (3) and (4):


(3) $$C=\Phi_{clustercoff}\;\left(M_{adj}\right)=\frac{1}{N}\overset{N}{\underset{i=1}{\sum }}C_{i}$$

(4) $$C_{i}=\frac{2e_{i}}{K_{i}\left(K_{i}-1\right)}$$

ei is the number of adjacent nodes of node I, Ci is the clustering coefficient of node I, and C is the CC of the corresponding region. Four adjacency matrices ND and CC corresponding to four motion tasks of the affected limb were calculated. In addition, the coherence of the M1 region of the contralesional hemisphere and the M1 region of the affected hemisphere (M1L or M1R) was calculated to observe its influence on selecting an appropriate treatment strategy for rTMS.

The four ND/CC values corresponding to the four repetitive tasks of the affected limb were averaged across the six regions and the entire test range. Seven mean ND/CC values corresponding to the six regions and the entire test range were obtained. The Coeff of the two M1 regions was averaged over the four repetitive motion tasks.

For the two fNIRS tests completed by each patient, the average increments of ND and CC across the test range were calculated. The average increment of two network parameters (NetPara_Inc) was calculated as motor function recovery (i.e., increase in FMA-UE score) to confirm the relationship between network parameters and motor functions.

Considering the relationship between network parameter mean increment NetPara_Inc and motor function, the NetPara_Inc was used to evaluate the therapeutic effect of rTMS. FMA-UE scores were not directly used to assess treatment outcomes because the range of total FMA-UE scores varied significantly among study patients. NetPara_Inc data were statistically analyzed to distinguish the therapeutic effect between TMS1 and TMS2 groups. In addition, the difference in NetPara_Inc between the two groups and the average NetPara_Inc in the rTMS1 group were used to determine whether the rTMS treatment was appropriate. In this study, the parameters corresponding to the four repeated tasks were used in the statistical analysis instead of the average due to the small number of patients.

Finally, the network parameters and Coeff characteristics were analyzed to determine the difference between rTMS treatment with good effect and rTMS treatment with poor effect. The following calculations were made.

1. Considering that the treatment regimen for rTMS was based on TMS information measured after the first fNIRS test, brain network parameters obtained from the first fNIRS test were analyzed. For ND and CC, differences between the M1-Healthy and the M1-Affected were calculated. Given that the treatment strategy of rTMS in the contralesional hemisphere was the focus of this study, we calculated and evaluated the corresponding difference ratios of ND and CC in two M1 regions (CC_DiffRatio_inH and ND_DiffRatio_inH). These ratios were more valuable than absolute differences in identifying the common characteristics of patients.
2. ND and CC were statistically analyzed in the M1-Healthy and M1-Affected to determine the suitability and appropriability of rTMS treatment. Coeff, CC_DiffRatio_inH, and ND_DiffRatio_inH of the two M1 regions. These data were expected to provide recommendations for rTMS treatment based on area network parameters.

### 2.4. Statistical analysis

The statistical software SPSS 22.0 was used to analyze the data. Measurement data were expressed as $\bar{X}\pm S$ , and the analysis of variance was used for intergroup comparison. The LSD (least significant difference) test was used for pair comparison, and the paired T-test was used for intragroup comparison. n was used for counting data, and the chi-square test was used for intergroup comparison. *p* < 0.05 indicated a significant difference.

All calculations and analyses except statistical analysis were performed using the Matlab R2014a (MathWorks, Inc.). The Anaconda3 spyder was used to analyze the statistical characteristics of two groups of data of different sizes. *p* < 0.01 indicated a significant difference.

## 3. Results

### 3.1. Case data results

The age, gender, affected side, and stroke type of all patients were comparable and had no significant difference (Table 2).

**Table 2:** Comparison of general data of the three group.

Group	Number	Sex (*n*)^a^		Age (years)	Hemiparesis (*n*)^b^		Type of stroke (*n*)		
		M	F		L	R	Infarction	Hemorrhage	Infarction + Hemorrhage
Control	15	12	3	57.20 ± 6.09	6	9	8	6	1
TMS1	9	5	4	60.00 ± 6.98	5	4	5	4	0
TMS2	9	7	2	59.56 ± 8.00	4	5	5	5	0
F/X^2^		1.761	1.523	0.583	1.623	1.563	0.523	0.554	0.642
P		0.415	0.512	0.564	0.805	0.722	0.762	0.758	0.834

a M, male; F, female.

b L, left; R, right.

### 3.2. Comparison of the upper limb motor function in three groups at different time points before and after treatment

**Figure 4:**
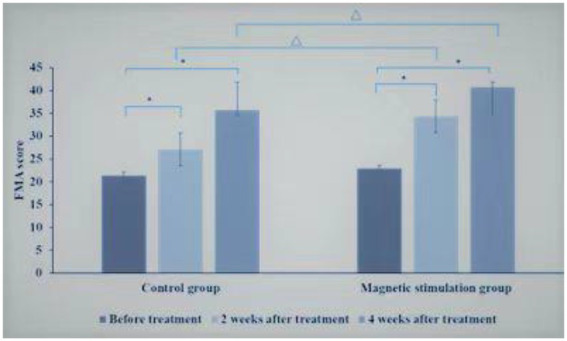
Comparison of FMA scores between control group and magnetic stimulation group. *p < 0.001; ∆*p* < 0.001.

Before treatment, there was no statistical difference in upper limb motor function score of Fugl-Meyer Assessment (FMA) scale between the control group and the magnetic stimulation group (*p* = 0.178). In 2 groups, compared with before treatment, the upper limb motor function score of Fugl-Meyer Assessment (FMA) scale increased significantly after 2 and 4 weeks of treatment, and the upper limb motor function score of FMA increased significantly after 4 weeks of treatment compared with 2 weeks of treatment (*p* < 0.01).Compared with the control group at the same time point, the FMA upper limb motor function score increased faster at 2 and 4 weeks of treatment in the magnetic stimulation group (*p* < 0.001),as shown in Figure 4.

**Figure 5:**
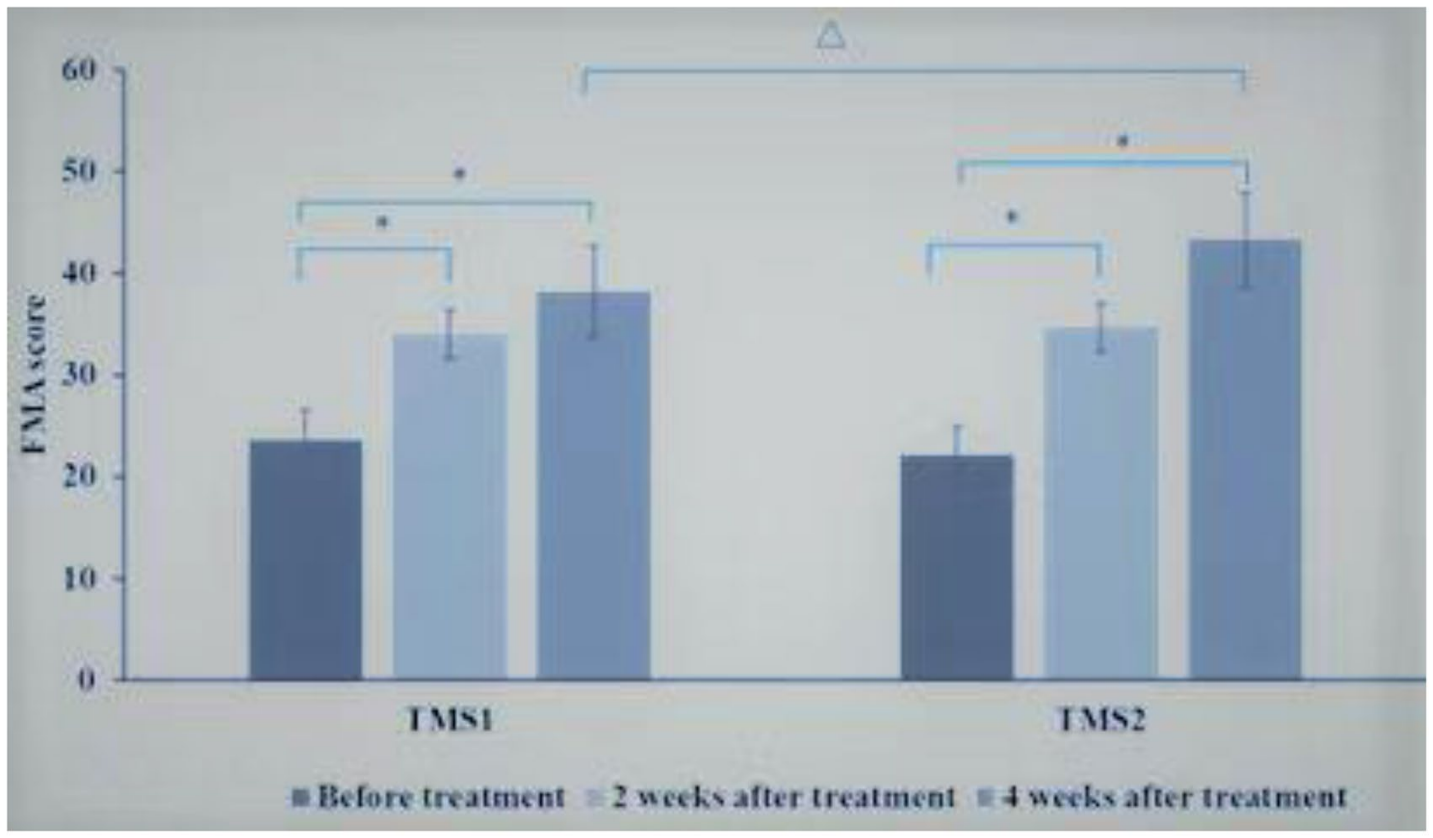
Comparison of FMA scores between TMS1 group and TMS2 group before and after treatment. *p < 0.001; ∆*p* = 0.010.

Comparison between TMS1 group and TMS2 group at the same time point, before treatment, there was no significant difference in FMA upper limb motor function score between TMS1 group and TMS2 group. After 2 weeks of treatment, there was no significant difference in FMA upper limb motor function score between TMS1 group and TMS2 group. After 4 weeks of treatment, the improvement of FMA upper limb motor function score in TMS2 group was more significant than that in TMS1 group (*p* = 0.01; Figure 5).

### 3.3. fNIRS test results

**Figure 6:**
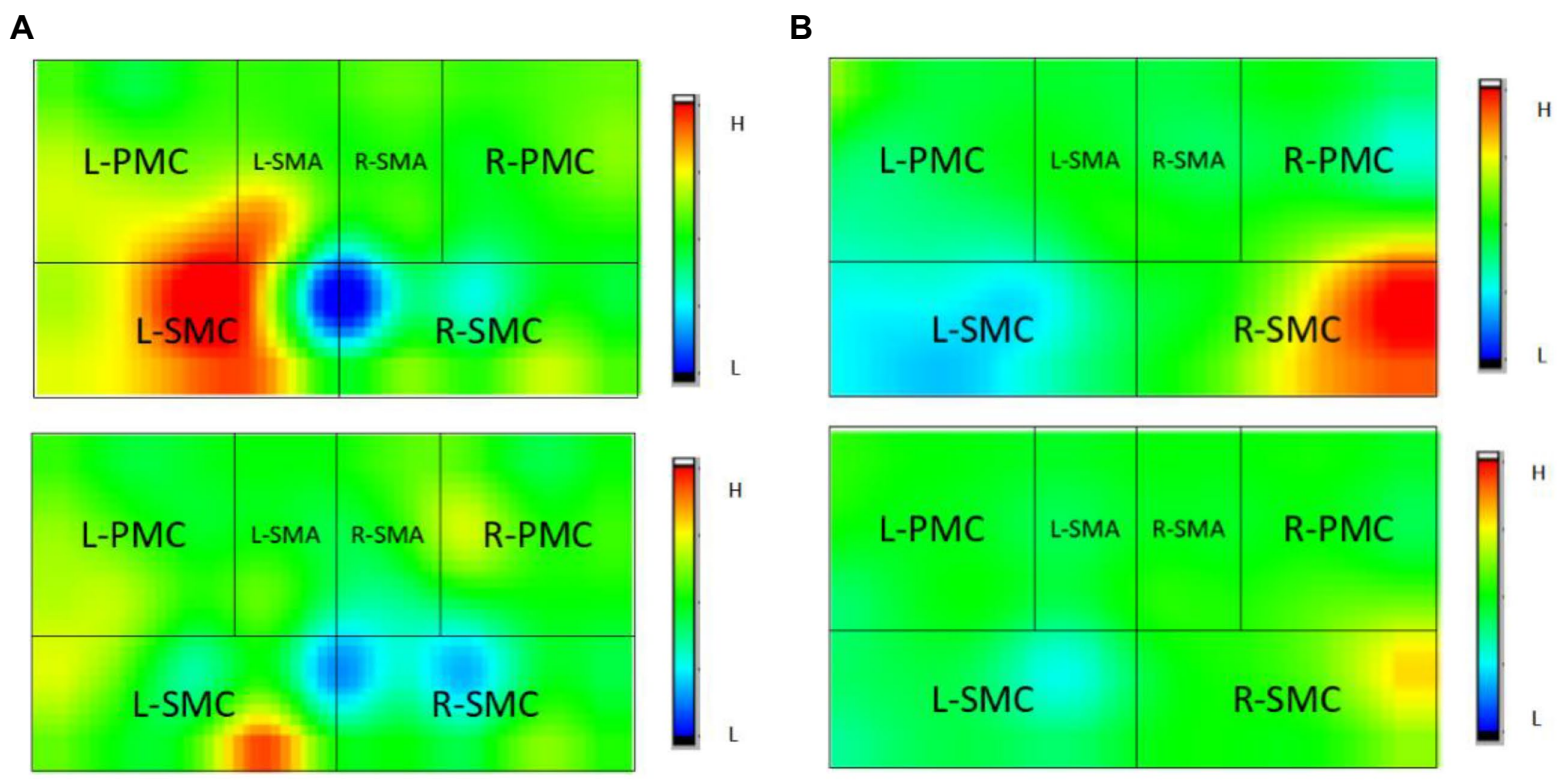
Observation of changes of HbO2 concentration in brain of some stroke patients: **(A)** Control group, **(B)** Magnetic stimulation group. H, high; L, low; L, left; and R, right. **(A)** A case of cerebral infarction in the right hemisphere. **(B)** A case of cerebral infarction in the left hemisphere.

**Figure 7:**
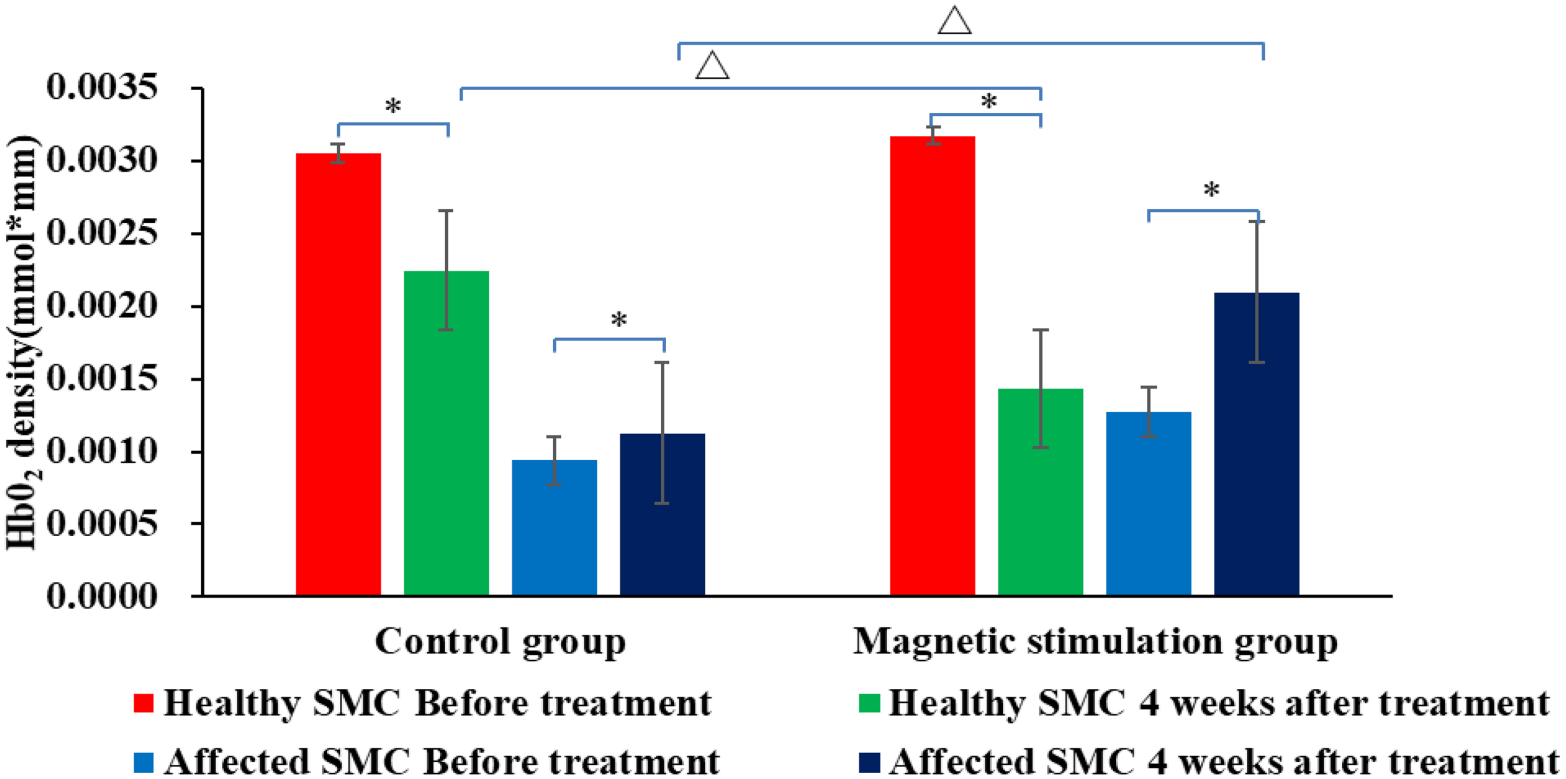
Comparison of HbO2 content in SMC area of the two groups before and after treatment. *p < 0.001; ∆*p* < 0.001.

Figures 6, 7 and Table 3 show that the HbO2 content in healthy sensory motor cortex (SMC) area of magnetic stimulation group and control group was higher than that in other region of interest (ROI) area before treatment. Tables 3, 4 and Figure 7 illustrate the comparison of ROI between the two groups. After 4 weeks of treatment, the HbO2 content in the SMC area of the healthy side was significantly decreased (*p* < 0.001), while the HbO2 content in the SMC area of the affected side was significantly increased (*p* < 0.001).The changes were more significant in the magnetic stimulation group.

**Table 3:** Comparison of HbO2 concentration in the unaffected hemisphere ROI in the three groups before and after treatment.

Group	Number	UH^a^ PMC		UH SMA		UH SMC	
		T0^b^	T2^c^	T0	T2	T0	T2
Control	15	0.001490 ± 0.000739	0.001422 ± 0.000592	0.001264 ± 0.000610	0.001261 ± 0.000666	0.003049 ± 0.000662	0.002246 ± 0.000406^a^
TMS1	9	0.001431 ± 0.000704	0.001135 ± 0.000654	0.001130 ± 0.000146	0.001248 ± 0.000286	0.003116 ± 0.000752	0.001524 ± 0.000869^a^
TMS2	9	0.001397 ± 0.000620	0.001247 ± 0.000503	0.001169 ± 0.000297	0.001037 ± 0.000238	0.003227 ± 0.000813	0.001338 ± 0.000614
F		0.054	0.715	0.282	0.645	0.167	7.382
P^d^		0.947	0.497	0.756	0.532	0.847	0.002

a UH, unaffected hemisphere.

b T0, before treatment.

c T2, after 4 weeks of treatment.

d ^*^p < 0.05 vs. control group, ^a^*p* < 0.05 vs. treatment group before treatment.

**Table 4:** Comparison of HbO2 concentration in the affected hemisphere ROI in the three groups before and after treatment.

Group	Number	AH^a^ PMC		AH SMA		AH SMC	
		T0^b^	T2^c^	T0	T2	T0	T2
Control	15	0.000999 ± 0.000461	0.000983 ± 0.000322	0.001122 ± 0.000438	0.001226 ± 0.000454	0.000938 ± 0.000160 0.001579 ± 0.000824	0.001125 ± 0.000232^a^
TMS1	9	0.001036 ± 0.000234	0.001039 ± 0.000229	0.001375 ± 0.000497	0.001264 ± 0.000501	0.001579 ± 0.000824^*^	0.002043 ± 0.000487^*^
TMS2	9	0.000964 ± 0.000391	0.001176 ± 0.000289	0.000997 ± 0.000182	0.000908 ± 0.000462	0.000965 ± 0.000233^#^	0.002147 ± 0.000574^a^
F		0.076	1.250	2.063	1.660	6.288	21.955
P^d^		0.927	0.301	0.145	0.207	0.005	< 0.001

a AH, affected hemisphere.

b T0, before treatment.

c T2, after 4 weeks of treatment.

d ^*^p < 0.05 vs. control group; ^#^*p* < 0.05 vs. TMS1 group; ^a^*p* < 0.05 vs. treatment group before treatment.

### 3.4. Relationship between activation of the dominant hemisphere and motor function

**Figure 8:**
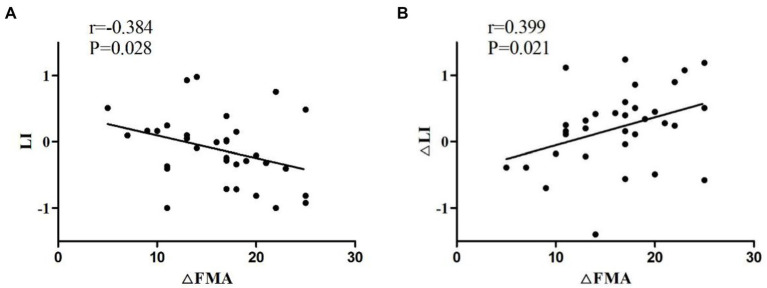
**(A)** Negative correlation of the LI of patients before treatment with ∆FMA. **(B)** Positive correlation of ∆LI with ∆FMA. ∆FMA, Mean increment of FMA-UE before and after 4 weeks of treatment; ∆LI, LI difference between before and after 4 weeks after treatment.

As shown in Figure 8, the Spearman correlation test was used to assess the relationship between LI and FMA-UE increment (∆FMA). LI before treatment was negatively correlated with ∆FMA (*r* = −0.384, *p* = 0.028). The change in LI after 4 weeks of treatment compared with that before treatment (∆LI) and ∆FMA were compared. As ∆FMA increased, ∆LI also increased (*r* = 0.399, *p* = 0.021; Figure 7).

### 3.5. Spatial positioning of rTMS

**Figure 9:**
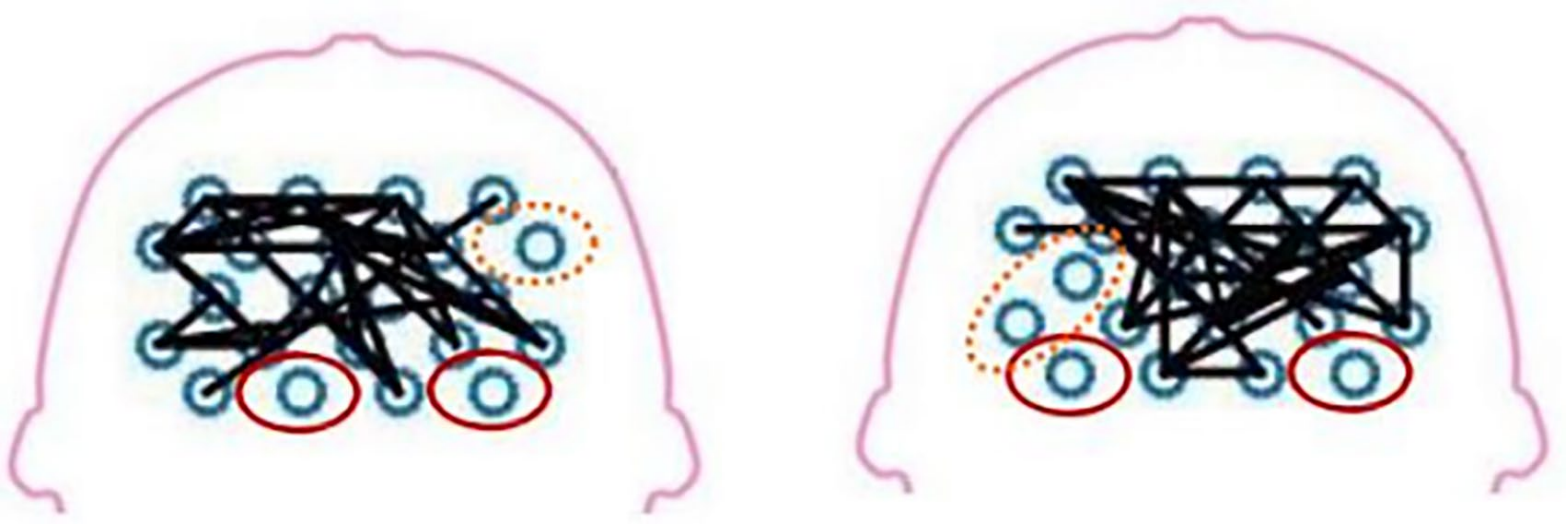
Network topologies of the test brain scope of two patients.

The fNIRS can detect the blood oxygen information to reflect the neural activity state of the corresponding brain region and construct the brain network. Figure 9 illustrates a schematic diagram of the network topology in the transverse axis of the brain range tested in two patients. Besides, the figure demonstrates that the intensity of brain network connection has been improved in SMC, PMC, and SMA. The areas of red solid coil mean the island areas. The island area refers to an isolated activated brain area that cannot transmit excitation to other related brain areas. Areas connected by solid black lines mean that brain scopes have stronger brain network connections. The areas circled by the orange dotted line are in between, with poor brain network connectivity. For further research, this study measured some brain network parameters and some indicators of motor function, then performed calculation of coherence analysis.

### 3.6. Relationship between network parameters and motor function

**Figure 10:**
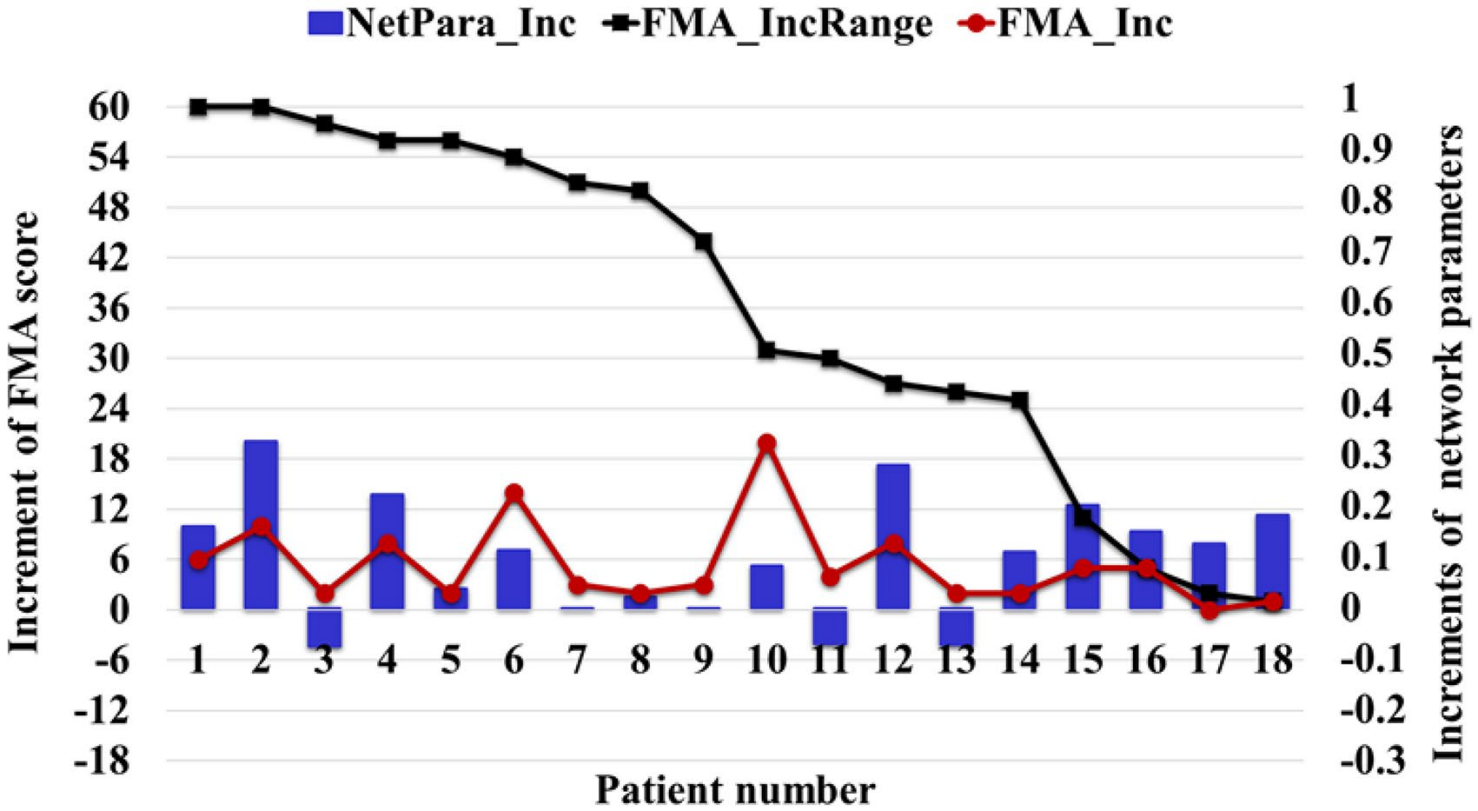
Relationship between the average increment of network parameters and FMA-UE score. The NetPara_Inc indicated the average increment of network parameters, FMA-UE_IncRange indicated the increment range of the FMA-UE score, and FMA-UE_Inc indicated the actual increment of the FMA-UE score.

The brain network parameters of 18 patients in the treatment group were analyzed, and the FMA’s upper limb motor function score was used to indicate motor function. The relationship between network parameters and motor function was studied by comparing the average increment of network parameters with the increment of FMA-UE score. About 83% (15/18) of patients’ network parameters increased with increasing FMA score, and only 6% (3/18) of patients’ FMA-UE increment was less than four points, corresponding to decreased network parameters (Figure 10). Network parameters increased with the recovery of motor functions.

### 3.7. Network parameter characteristics of different rTMS treatment regimens

**Figure 11:**
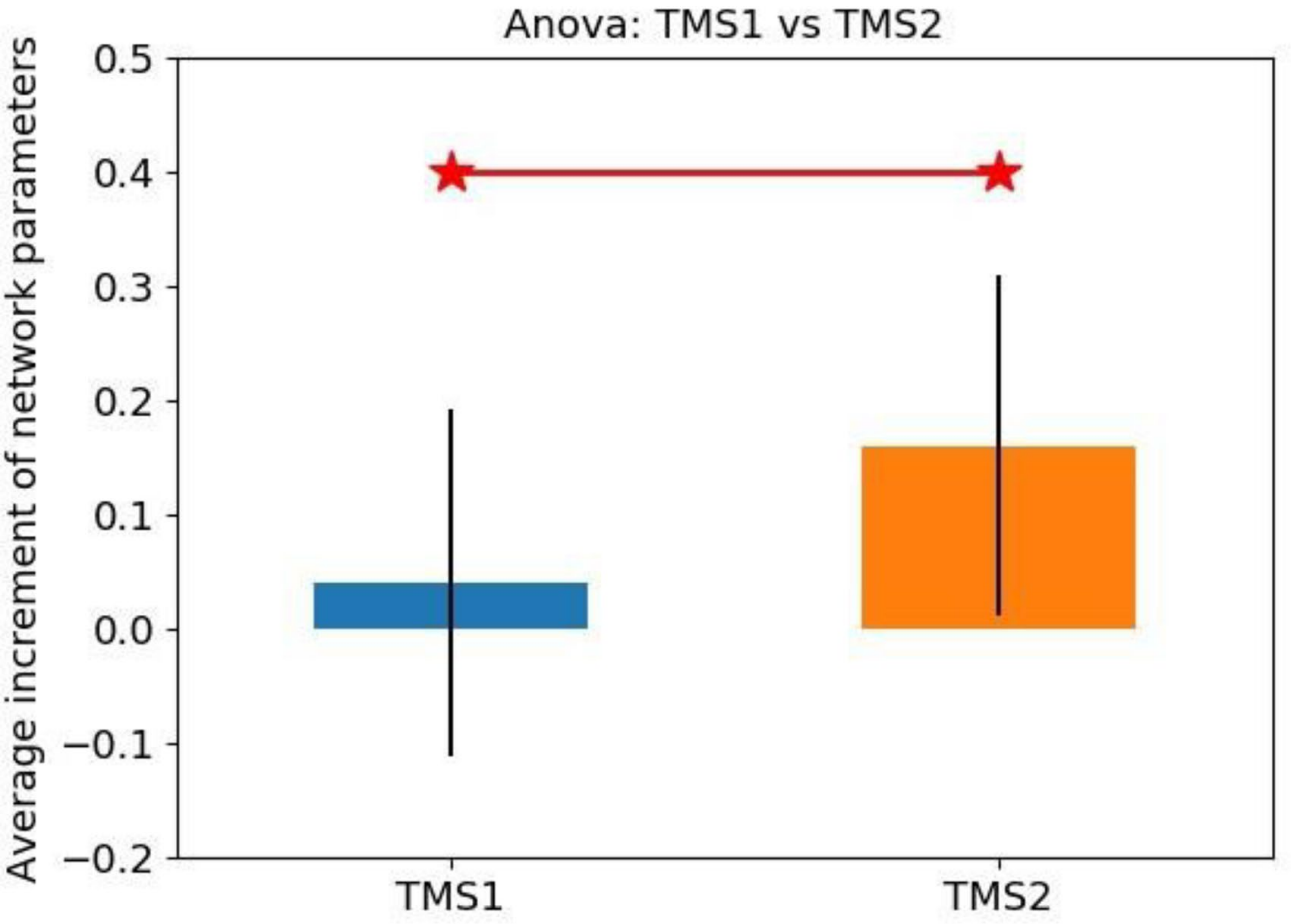
Statistical analysis of the average increment of network parameters.

The statistical analysis results of network parameter mean increment for TMS1 and TMS2 groups are shown in Figure 11. The NetPara_Inc values of TMS1 and TMS2 groups were 0.040 and 0.160 mM·mM, respectively. Considering that patients had a certain self-recovery ability, when NetPara_Inc was less than 0.04 mM·mM, the rTMS treatment effect was poor, which was the average value of NetPara_Inc in the TMS1 group. When NetPara_Inc >0.04 mM·mM, rTMS had improved therapeutic effect.

(NetPara_Inc) of the two rTMS processing schemes. NetPara_Inc indicates the average increment of network parameters. “TMS1” refers to the promotion of magnetic stimulation to the M1-Affected, whereas inhibitory magnetic stimulation treatment to the M1-Healthy, “TMS2” refers to the promotion of magnetic stimulation to the M1-Affected.

**Figure 12:**
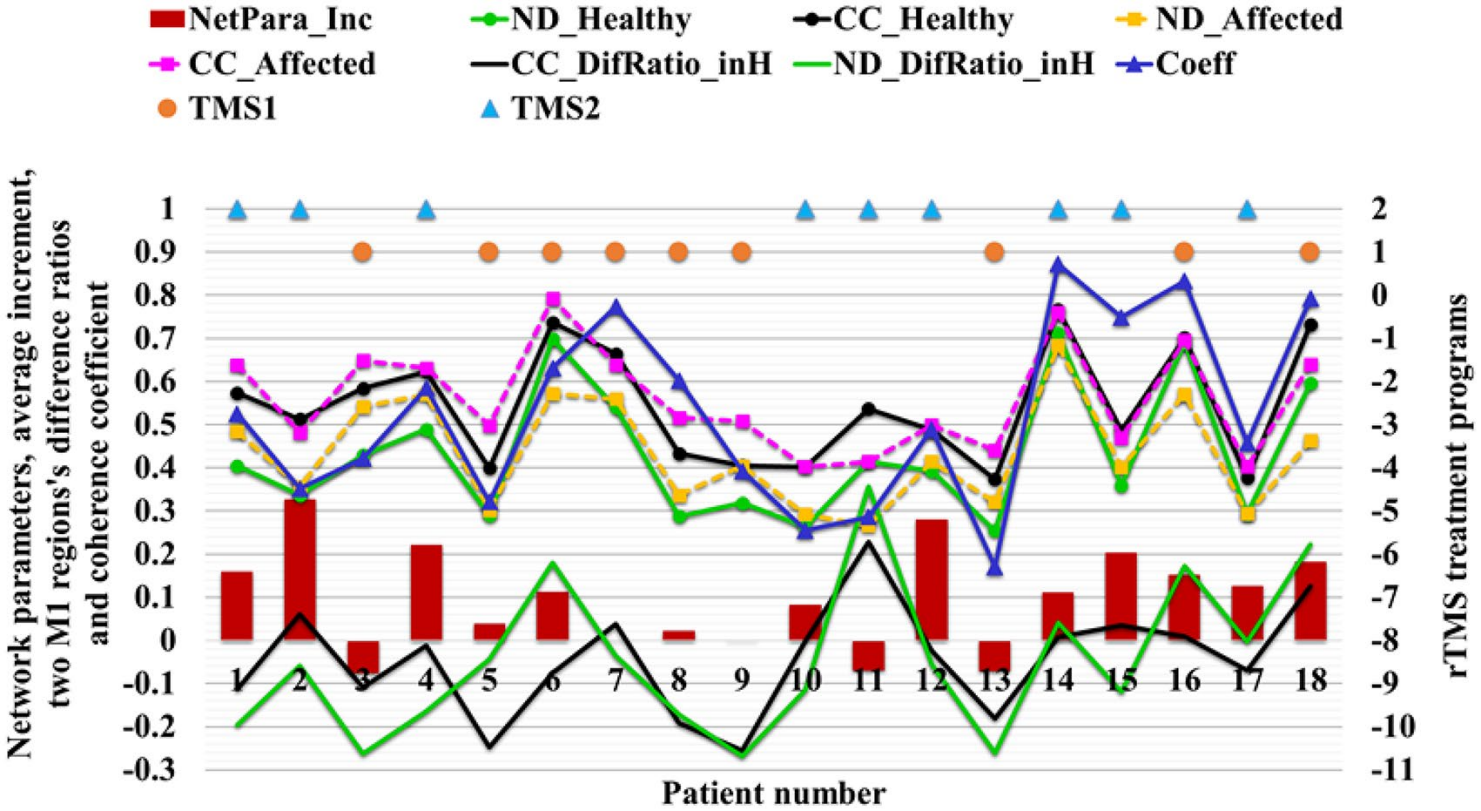
Relationship between network parameters and rTMS treatment strategy.

In the TMS1 group, only three patients (i.e., patients 6, 16, and 18) had NetPara_Inc higher than 0.04 mM·mM. Six other patients showed little or no increase in NetPara_Inc. NetPara_Inc was elevated in eight patients in the TMS2 group and not in one patient (patient 11). As shown in Figure 12, a significant difference in incremental NetPara_Inc was observed between the two groups (*X*^2^ = 5.844, *p* = 0.016).

This result indicated that the stimulative effects in the M1-Affected could produce better therapeutic effects than the combined effects of the inhibitory stimuli in the M1-Healthy and the stimulative stimuli in the M1-Affected (Table 5).

**Table 5:** Difference of the average increment of network parameters between TMS1 and TMS2 groups.

Group	Incremental or not^a^		Total	X^2^	P
	Y	N			
TMS1	3	6	9	5.844	0.016
TMS2	8	1	9		
Total	11	7	18		

a Y, yes; N, not.

“NetPara_Inc” indicates the average increment of network parameters, “ND” indicates the node degree, “CC” indicates the clustering coefficient, “Healthy” and “Affected” indicates the corresponding M1 region, and “DifRatio_inH” indicates the ratio of differences between two M1 regions. “Coeff” represents the Coeff of the two M1 regions. “TMS1” refers to promoting magnetic stimulation in the M1-Affected and inhibiting magnetic stimulation in the M1-Healthy, and “TMS2” refers to promoting magnetic stimulation in the M1-Affected.

## 4. Discussion

### 4.1. Relationship between laterality index and motor function

According to the study results, the motor recovery in patients with nonlesional hemispheric dominance before treatment is superior to that in patients with lesional hemispheric dominance, which may be associated with good corticospinal tract integrity (Stinear et al., 2007; Wang et al., 2014). However, the trend of LI after treatment shows that LI values gradually change from negative to positive with the recovery of motor function, suggesting a gradual transition to bilateral or affected hemispheric activation, which may conflict with the view of interhemispheric inhibition (Kuo et al., 2020). Not all stroke patients uniformly show severe interhemispheric inhibition (Yamada et al., 2013). Tamashiro indicated that some patients have not shown profound interhemispheric inhibition until after the acute phase.. Moreover, LI, as an indicator of motor function recovery, has certain limitations and may be affected by the ceiling effect (Tamashiro et al., 2019). Therefore, authors prefer to use brain network connection parameters to predict motor recovery.

### 4.2. Relationship between network parameters and motor function

About 83% of patients experience increased network parameters as motor function scores increase, suggesting a positive correlation between the average increment of network parameters and motor function. This finding is consistent with the conclusions of previous studies (Kalinosky et al., 2017; Schlemm et al., 2020; Sotelo et al., 2020; Zhai et al., 2020). However, some cases have not fitted the positive correlation. The possible reasons are the individual differences among different patients and the unavoidable subjective factors in evaluating upper limb motor function by physicians in FMA-UE (Kristersson et al., 2019).

According to the results of previous and current studies, the increase and decrease of brain network parameters can reflect the enhancement and weakening of motor function, respectively. Given the small sample size of this study, the specific mathematical relationship between network parameters and motor function level needs to be further verified by analyzing data from a large number of patients.

### 4.3. Network parameter characteristics of different rTMS treatment strategies

The therapeutic effect of brain network parameters and rTMS show that the absolute value of network parameters (especially the ND) in left and suitable M1 regions and the proportion of differences between the two regions (M1_Healthy and M1_Affected) can be directly used in the selection of treatment options for rTMS. When the ND of M1-Healthy is significant, ND_Healthy >0.52. When the ND of M1-Healthy is significantly higher than that in the affected hemisphere (ND_DifRatio_inH > 0.13), M1-Healthy may be inhibited, and M1-Affected may be promoted simultaneously. When the ND of the M1 region of the contralesional hemisphere is low (ND_Healthy <0.52), the NDs of the two M1 regions are not significantly different.

Therefore, performing inhibitory magnetic stimulation in the M1-Healthy is not recommended. Instead, stimulatory magnetic stimulation in the M1-Affected alone is suggested.

A previous study found that inhibition in the contralesional hemisphere depends on corticospinal tract injury in the affected hemisphere (Du et al., 2019). If the brain injury is mild and sufficient structural reserve is present at the injury site, inhibitory stimulation of the contralesional hemisphere can produce improved results. In this case, the connectivity between the two M1 regions is likely to be good if the functional brain nodes have good connectivity and communication capacity. Therefore, an extensive area of network parameters can be obtained. Additionally, this study found that if the brain is severely damaged and the structural reserve of the affected hemisphere is insufficient, the inhibitory stimulation of the contralesional hemisphere leads to poor therapeutic effects (Stinear et al., 2007). Therefore, if the connectivity and communication capacity of functional nodes in the brain are poor, the connectivity between the two M1 regions may be poor, and a small area of network parameters can be obtained. Therefore, our results are consistent with previous studies (Stinear et al., 2007).

In addition, our study proposes the relative relationship between parameters of two M1 regional networks as another means to guide rTMS processing. When the network parameters of M1-Healthy are larger than those of the M1-Affected, the neural activity of the healthy side hemisphere is higher than the affected side hemisphere. Results show that a solid interhemispheric inhibition in the stroke hemisphere is noted in the stroke hemisphere. In this case, the inhibition of the healthy M1 region is recommended. By contrast, when the network parameters in the M1-Healthy are smaller than those in the M1-Affected, the inhibitory stimulus in the M1-Healthy may disrupt the balance of interhemispheric competition. Therefore, suppression is not recommended.

Furthermore, if the network parameters of the two regions are small, the network parameters of the M1-Healthy are still much larger than those of the M1-Affected (patient 11), then the inhibition of stimulus in the M1-Healthy should also be considered. The results of patient 11 suggest that the difference ratio of network parameters in the M1 region on both sides may play a vital role in guiding rTMS treatment. However, in our study, only patient 11 has this outcome. Therefore, further validation in other patients is required.

Our study covered patients with different types, lesion sites, and disease degrees. Therefore, this method has universality and can be used to guide the treatment of other neurological diseases. However, the threshold proposed in this study is based on data from 18 patients only. By analyzing a large number of datas from many patients, an accurate mathematical model can be obtained to give precise thresholds of absolute value and difference ratio.

## 5. Conclusion

In conclusion, by comparing the brain network parameters and FMA-UE score of patients with stroke, this preliminary study found that rTMS treatment is beneficial to the recovery of the upper limb motor function of patients with stroke, significantly improves the intensity of brain network connection, and reduces the intensity of the island area. In consideration with the brain functional network connection states, the corresponding adjustment should be made to the treatment plan of rTMS to achieve an optimal therapeutic effect and precise rehabilitation treatment. When the ND of the M1_Healthy region is less than 0.52, performing promotion therapy only in the affected hemisphere is suggested. When the ND of the M1_Healthy region is more significant than 0.52 and much larger than that in the M1_Affected region, inhibition in the contralesional hemisphere and high-frequency excitatory magnetic stimulation in the affected hemisphere can be performed.

## Data availability statement

The original contributions presented in the study are included in the article/supplementary material, further inquiries can be directed to the corresponding authors.

## Ethics statement

The studies involving human participants were reviewed and approved by Suzhou Dushu Lake Hospital Ethics Committee. The patients/participants provided their written informed consent to participate in this study. Written informed consent was obtained from the individual(s) for the publication of any potentially identifiable images or data included in this article.

## Author contributions

JN and WJ contributed equally to this manuscript. XG, YF, HQ, JD, and JZ prepared the manuscript and performed the experiments. HW, CL, and MS supervised the project and edited the manuscript. All authors contributed to the article and approved the submitted version.

## Funding

This work was supported in part by National Natural Science Foundation of China (No. 81672244), Suzhou Minsheng Science and Technology Key Technology Application Research project in 2019 (Project No.: SS2019051), and General Projects of Jiangsu Provincial Natural Fund (Grant No. SBK2021020630).

## Conflict of interest

The authors declare that the research was conducted in the absence of any commercial or financial relationships that could be construed as a potential conflict of interest.

## Publisher’s note

All claims expressed in this article are solely those of the authors and do not necessarily represent those of their affiliated organizations, or those of the publisher, the editors and the reviewers. Any product that may be evaluated in this article, or claim that may be made by its manufacturer, is not guaranteed or endorsed by the publisher.
